# Breathing Rate Estimation from Head-Worn Photoplethysmography Sensor Data Using Machine Learning

**DOI:** 10.3390/s22062079

**Published:** 2022-03-08

**Authors:** Simon Stankoski, Ivana Kiprijanovska, Ifigeneia Mavridou, Charles Nduka, Hristijan Gjoreski, Martin Gjoreski

**Affiliations:** 1 Emteq Ltd., Brighton BN1 9SB, UK; ivana.kiprijanovska@emteqlabs.com (I.K.); ifi.mavridou@emteqlabs.com (I.M.); charles@emteqlabs.com (C.N.); hristijang@feit.ukim.edu.mk (H.G.); 2 Faculty of Electrical Engineering and Information Technologies, Ss. Cyril and Methodius University in Skopje, 1000 Skopje, North Macedonia; 3 Faculty of Informatics, Università della Svizzera Italiana, 6900 Lugano, Switzerland; martin.gjoreski@usi.ch

**Keywords:** breathing rate, machine learning, PPG, VR headset, motion artifact removal, information fusion

## Abstract

Breathing rate is considered one of the fundamental vital signs and a highly informative indicator of physiological state. Given that the monitoring of heart activity is less complex than the monitoring of breathing, a variety of algorithms have been developed to estimate breathing activity from heart activity. However, estimating breathing rate from heart activity outside of laboratory conditions is still a challenge. The challenge is even greater when new wearable devices with novel sensor placements are being used. In this paper, we present a novel algorithm for breathing rate estimation from photoplethysmography (PPG) data acquired from a head-worn virtual reality mask equipped with a PPG sensor placed on the forehead of a subject. The algorithm is based on advanced signal processing and machine learning techniques and includes a novel quality assessment and motion artifacts removal procedure. The proposed algorithm is evaluated and compared to existing approaches from the related work using two separate datasets that contains data from a total of 37 subjects overall. Numerous experiments show that the proposed algorithm outperforms the compared algorithms, achieving a mean absolute error of 1.38 breaths per minute and a Pearson’s correlation coefficient of 0.86. These results indicate that reliable estimation of breathing rate is possible based on PPG data acquired from a head-worn device.

## 1. Introduction

A by-product of affective computing research for real-world applications has been the exploration of improved methodologies, sensor technologies, and computational approaches [1,2]. Such advances allow for the reliable detection of various physiological signals, such as heart rate. Nowadays, these are provided as part of novel wearable devices [3] that can be applied in different settings, including virtual reality (VR). The benefits of utilizing affective computing approaches via wearable sensors include, but are not limited to, improving human-computer interactions and assisting in the development of healthcare and wellbeing interventions.

Monitoring breathing activity is an essential component of affective computing approaches. Changes in breathing rate have been explored as an index of clinical deterioration [4], anxiety [5], and affective states [6]. Increased breathing rate is also associated with stress, and negative and high arousing experiences [7]. Approaches for monitoring breathing rate include a chest strap that monitors chest movement and abdomen movements (e.g., NUL-236 [8]), a finger-clipped pulse oximeter (e.g., Renesas OB1203SD-RL-EVK [9]), or a face-worn breathing mask [10,11] While these devices provide accurate measures, their usage is mostly limited to laboratory environments

Given that the monitoring of cardiac activity is less challenging than the monitoring of breathing, e.g., using photoplethysmography (PPG) or electrocardiogram (ECG) sensors incorporated in chest- or wrist-mounted devices, a variety of algorithms have been developed to estimate breathing activity from heart activity. However, there is still no thorough comparison between the accuracy of the breathing rate extracted using ECG/PPG-based approaches and the classical systems that are monitoring chest movement.

**Figure 1:**
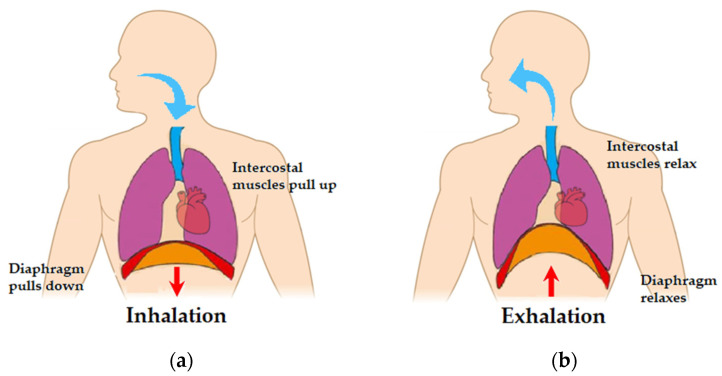
Heart, lungs, and diaphragm during: (**a**) inhalation; (**b**) exhalation. Illustration based on magnetic resonance imaging (MRI) of natural chest movement [13].

The overall respiration control system consists of a network of neurons in cortex and medulla/pons that exert voluntary control and automatic control. Spontaneous respiration is produced by rhythmic discharge of motor neurons that innervate the respiratory muscles. One physiological relation between breathing and heart activity, often utilized by breathing rate estimation algorithms, is the following:

- During inhalation (Figure 1a), the respiratory neurons regulate the activity of the respiratory muscles. One of them is the diaphragm, which plays a major role in breathing control. In fact, during inhalation, it moves downward and makes more room in the chest. This causes the heart volume to increase. The sinoatrial node informs the central nervous system (CNS) of the increased heart volume, and, in turn, the CNS sends a signal back to the heart to increase the heart rate [12].
- During exhalation (Figure 1b), this process is reversed. That is, the diaphragm relaxes and shrinks the room in the chest, resulting in a decrease in heart volume. The CNS, informed about the decreased heart volume by the sinoatrial node, sends a signal to the heart to decrease the heart rate.

Thus, during inhalation, the heart rate increases; during exhalation, the heart rate decreases. Consequently, by continuously monitoring each heart bit and the corresponding peak-to-peak (P-P) intervals, i.e., the duration between two consecutive heart bits, can be estimated when a subject is inhaling and exhaling. Such P-P intervals, for example, can be extracted from PPG data.

Even though the relation between heart and breathing activity is well known, estimating breathing rate from heart rate outside of a laboratory setting is still a challenge, which is why consumer fitness devices are rarely providing breathing rate estimations, even though most of them incorporate a PPG sensor. The challenge is even bigger when new wearable devices with novel sensor placement are used. The specific device used in this study is a head-worn VR mask equipped with a PPG sensor, which is placed on the forehead of a subject. To tackle this challenge, we designed a novel breathing rate estimation algorithm based on advanced signal processing and machine learning (ML) techniques. The algorithm takes as input 20-s PPG data, which are filtered, and motion artifacts are removed from them. The filtered PPG data are then used to detect the P-P intervals. Representative features are calculated from both the P-P intervals series and the filtered PPG data. The features are then fed to a regression ML model, which outputs the estimated breaths per minute from the input window. The proposed algorithm is compared to existing approaches from the related work using two separate datasets.

The key contributions of the work presented in this paper can be summarized as follows:

- Preparation of two annotated datasets containing PPG data from a total of 37 subjects, intended to be used for evaluation of the performance of breathing rate estimation algorithms. The data were acquired using a head-mounted PPG sensor (centered on the forehead). We provide public access to one of the datasets (13 subjects).
- A novel ML-based method for estimation of breathing rate from PPG data.
  (a) The method utilizes the correspondence between PPG and breathing rate by extraction of representative features from P-P time-series, including pulse rate variability (PRV) features, and various PPG-derived signals, some of which have not been used before by other research groups. It uses a window size of 20 s, which is shorter than state-of-the-art approaches, making our algorithm more responsive to physiological changes.
  (b) The method includes a novel scheme for removal of motion artifacts from the PPG data, as well as an efficient signal quality assessment procedure that identifies low-quality signals and rejects data unfit for accurate breathing rate estimation.
  (c) To the best of our knowledge, this is the first study that employs an ML-based method that estimates breathing rate from PPG data acquired from a head-worn device (VR headset).
- An extensive evaluation of the method is carried out here, including: (i) a comparison of our proposed method with three state-of-the-art benchmark/reference approaches; (ii) an analysis of the effects of changing the window size on its performance in terms of mean absolute error (MAE) and Pearson correlation coefficient (PCC); (iii) a comparison of the results obtained using different feature subsets; (iv) an analysis of the features’ importance and their contribution to the ML model’s predictions using the Shapley additive explanations (SHAP) method.

The remainder of the paper is organized as follows: In Section 2, we discuss the existing approaches for breathing rate estimation from PPG signals. In Section 3, we present the details on our collected datasets used for the development of the breathing rate algorithm. In Section 4, we describe the developed ML methodology for real-time breathing rate estimation from head-worn PPG sensor signals. Section 5 describes the evaluation setup and the comparison (baseline) methods used in the study. The evaluation results are presented and discussed in Section 6. Section 7 concludes the paper and provides recommendations for future work.

## 2. Related Work

In this section, we present a summary of existing breathing rate algorithms that utilize cardiac activity to estimate breathing rate, focusing on those based on PPG data.

**Figure 2:**
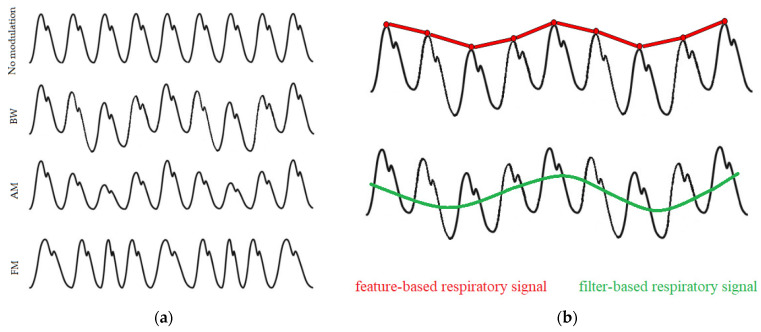
(**a**) PPG signals are subject to three respiratory modulations: baseline wander (BW), amplitude modulation (AM), and frequency modulation (FM); (**b**) Comparison of feature-based (red) and filter-based (green) techniques for extraction of exemplary respiratory signals.

Breathing activity may cause the PPG signals to modulate in three main ways, as illustrated in Figure 2a. These are: baseline wander (BW) modulation, which is influenced by changes in artery vasoconstriction and intrathoracic pressure throughout the breathing cycle; amplitude modulation (AM), which reflects changes in intrathoracic pressure and stroke volume during inhalation; and frequency modulation (FM), also known as the respiratory sinus arrhythmia (RSA), which causes the heart rate to increase during inhalation and to decrease during exhalation [14]. Signals dominated by these respiratory modulations can be extracted using various techniques, which mainly fall into two categories: filter-based or feature-based techniques (Figure 2b). Filter-based techniques consist of filtering the raw PPG signal to attenuate non-breathing frequency components. Feature-based techniques consist of extracting beat-by-beat feature measurements from the PPG signal.

Most breathing rate algorithms estimate the breathing rate by analyzing a window of these respiratory signals derived from the PPG data using various signal processing techniques. These techniques act in either the time or frequency domain. Time-domain techniques are focused on detection of individual breaths. The breathing rate is then estimated through the mean duration of the breaths detected in a previously defined window of data. These techniques typically detect individual breaths through peak and trough detection in the respiratory signals using adaptive thresholding methods [15,16]. One such method is presented by Schäfer et al. [17]. They propose a method based on counting prominent oscillations in the PPG data originating from breathing activity. The method includes detrending of respiratory signals derived from the PPG data, detection of peaks and troughs in the derived signals, and eventually applying heuristic rules to identify valid breaths. These rules are based on the idea that distinct breathing cycles are not characterized by the position of their maxima, but rather by the amplitude of the corresponding oscillation. Their results show that the FM of young subjects yields good approximations of mean breathing rate when using PPG segments longer than 60 s. Frequency-domain techniques, on the other hand, are focused on identifying the frequency component related to breathing in the respiratory signals. Such techniques most commonly entail spectral analysis of the frequencies contained in the respiratory signals. The spectral analysis consists of identifying the breathing frequency from a power spectrum calculated mainly using: fast Fourier transform (FFT) technique [18,19,20] and auto-regressive modelling [21,22]. In general, when using spectral analysis-based methods, the breathing rate is identified as the frequency corresponding to the spectral peak with the greatest magnitude in the range of 0.1–0.5 Hz, which is associated with spontaneous breathing rates. The use of frequency analysis-based methods has been extensively demonstrated. Karlen et al. [23] proposed a method for estimating the breathing rate from PPG signals. They derive three respiratory signals (BW, AM, FM) from the PPG signals using an incremental-merge segmentation algorithm and analyze the frequency content of each derived signal using the FFT technique. They experimented with window sizes of 16, 32, and 64 s and observed a positive trend for a lower error rate in larger windows. Their results also showed that the combination of the three derived respiratory signals contributes to a more robust estimation of breathing rate compared to individual estimation methods, achieving a root mean square error (RMSE) of 3 breaths per minute (bpm) for 32-s windows. Garde et al. [24] proposed an algorithm based on correntropy spectral density function (CSD) applied to the PPG data, which estimates the breathing rate by detecting the maximum frequency peak within the breathing frequency band. They investigated two window sizes of 60 s and 120 s and showed that longer windows significantly decrease the estimation errors; they achieved an RMSE of 4.2 bpm when using 60-s windows and 1.9 bpm when using 120-s PPG segments. Auto-regressive modelling has also been used to calculate the power spectral density (PSD) and identify the frequency contained within a respiratory signal. Shah et al. [25] used auto-regressive modelling to estimate child breathing rates during evaluation in the emergency department from PPG segments contaminated with movement artifacts. They achieved an MAE of 5.2 bpm for the age group of 5–12 years. Pimentel et al. [22] also used multiple auto-regressive models of different orders to determine the dominant breathing frequency in three respiratory signals (BW, AM, FM) derived from PPG signal. Their method was tested on two datasets collected in different clinical settings, achieving an MAE of 1.5 bpm and 4.0 bpm using a window size of 32 s.

Even though ML has already infiltrated many domains of health informatics [26], its efficiency in the field of breathing rate estimation from wearable sensors data has not been thoroughly explored; studies that have attempted to estimate breathing rate from PPG data using ML have been scarcely published. Shuzan et al. [27] proposed an ML method for breathing rate estimation from PPG data based on Gaussian process regression. They extracted several statistical and time-domain features from pre-processed PPG signals, with a window size of 32 s, as well as from their first and second derivatives. Their method was evaluated on data from 39 subjects collected during a resting period and achieved an MAE of 1.97 bpm using 5-fold cross-validation. Bian et al. [28] proposed an end-to-end deep learning (DL) method based on convolutional neural network architecture for breathing rate estimation from PPG data. Their DL architecture used only raw PPG signals as input, segmented with a window size of 60 s. Even after optimizing the hyperparameters of the DL architecture, their method achieved an MAE of 3.8 ± 0.5 bpm on data acquired in a clinical context using 5-fold cross validation, when only real data was used for training. The inclusion of synthetic data in the training process, however, decreased the MAE to some degree, to 2.5 ± 0.6 bpm.

In this study, we propose a novel breathing rate estimation method for extracting the breathing rate from PPG signals, based on advanced signal processing and ML techniques. The main motivation for using an ML-based approach for breathing rate estimation was its ability to fuse easily multiple representations of the breathing information encoded in PPG signals. Filter-based approaches usually focus on a single breathing representation and, as a result, they often fail to provide reliable estimation when the information is not clearly visible in the signal. Researchers have even made efforts to fuse multiple respiratory signals without using ML methods [29,30]; however, these methods require extensive tuning in order to work in different situations. Additionally, the availability of annotated datasets offers the possibility to develop and test adaptive and ersonalized ML models, which usually show better results compared to general rule-based approaches. To overcome the limitations of the filter-based approaches and to automatically learn the most relevant respiratory representation in various situations, our method utilizes the correspondence between breathing activity and heart activity by extraction of novel representative features from multiple PPG-derived signals. The extracted features are used to train a regression model that extracts a mean breathing rate from an input window. To the best of our knowledge, this is the first study that employs an ML method based on data from a PPG sensor mounted on a head-worn device.

## 3. Data

**Figure 3:**
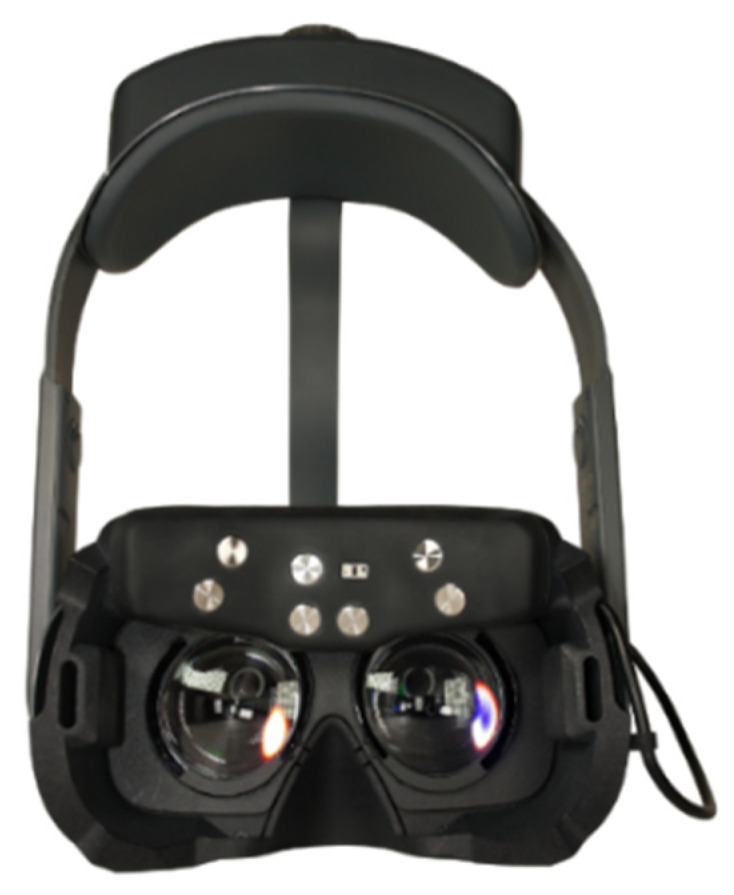
The emteqPRO Mask installed into the Pico Neo 2 Eye Virtual Reality (VR) headset.

For development and evaluation of the breathing rate estimation algorithm, we collected data from a total of 37 subjects during controlled breathing protocols. Ethical approval was obtained from the Bournemouth University Ethics Committee on 30 November 2020 (approval no. 33494). As per ethical requirements, all subjects also provided written informed consent before participating in the study. For data collection, we used the emteqPRO system [31,32], consisting of a VR sensor mask insert with a Pico Neo 2 Eye VR headset (Figure 3).

Each subject was instructed on how to wear the VR headset properly and we ensured the fit and the comfort of it prior to the start of the data collection procedure. From the biometric sensors incorporated in the emteqPRO system, we used only the data provided from the PPG sensor (centered on the forehead) and the accelerometer sensor. The PPG sensor, embedded within the emteqPRO mask, is a reflective type of PPG sensor. It has a high-intensity green light-emitting diode (LED) that sends light into the tissue and records how much light is reflected back to the diode, thus, measuring the expansion and contraction of capillaries based on blood volume changes. During the data collection procedure, PPG and accelerometer data were continuously recorded at fixed rates of 25 Hertz (Hz) and 50 Hz, respectively.

We conducted two data collection procedures, which resulted in two datasets:

- **Dataset 1:** This dataset includes data from 27 healthy subjects, 17 males and 10 females, with a mean age of 35 ± 14.2 (range 16–68). All the subjects followed breathing instructions via visual cues. Each subject completed one breathing session, containing breath cycles with different durations: small breaths of 3 s (10 repetitions), medium breaths of 5 s (8 repetitions), and large breaths of 8 s (6 repetitions), in that specified order. The total duration of the session was 2 min and 20 s, including 1-s breaks between each inhalation and exhalation. The total size of this dataset in minutes is 63 min.

**Figure 4:**
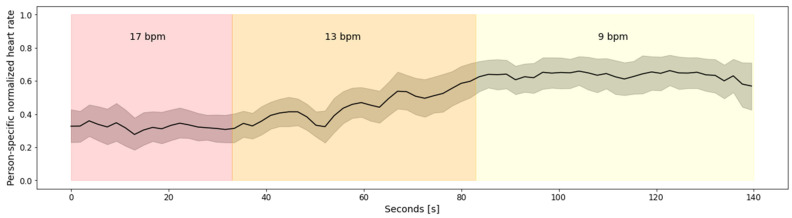
Aggregated heart rate for 27 subjects from Dataset 1. The increase in the heart rate corresponds to the increase in the duration of the guided breaths performed by the subjects.

Figure 4 depicts aggregated heart rate for all 27 subjects from Dataset 1. To avoid subject-specific differences, the heart rate was normalized using person-specific min-max normalization. The figure clearly depicts the relationship between the heart rate and the breathing rate, captured via emteqPRO. Namely, the heart rate is lower at the beginning of the breathing session, which corresponds to higher breathing rates—17 breaths per minute (bpm), on average, in the small breaths (duration of 3 s) scenario. As the breathing session progresses, the heart rate values rise. The highest heart rate values can be observed towards the end of the breathing session, which corresponds to the lower breathing rates (9 bpm on average) in the large breaths (duration of 8 s) scenario.

- **Dataset 2:** This dataset includes data from 13 healthy subjects, 7 males and 6 females, with a mean age of 28.8 ± 12.9 (range 20–59). All the subjects followed breathing instructions via audio cues, i.e., they were synchronizing their inhaling and exhaling with the different sound present in guided audio they were listening to. The dataset is divided into two parts:
  (a) Data from 3 subjects who completed five breathing sessions with a constant rate of 8, 12, 18, 12, 8 bpm, in that specified order. Each session lasted for one minute. There was a break between sessions of 1 min.
  (b) Data from 10 subjects who completed five breathing sessions with a constant rate of 12, 14, 16, 14, 12 bpm, in that specified order. Each session lasted for two minutes. There was a break between sessions of 1 min.

The total duration of this dataset in minutes is 115 min.

**Figure 5:**
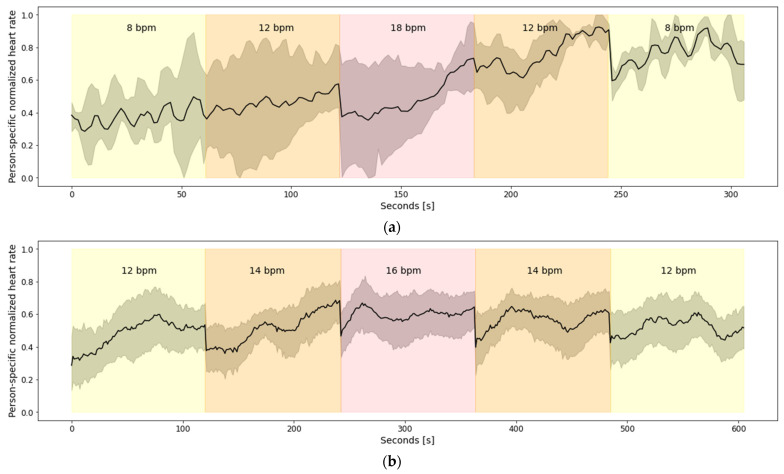
Aggregated heart rate for: (**a**) 3 subjects in Dataset 2.a; (**b**) 10 subjects from Dataset 2.b.

Figure 5a depicts aggregated heart rate for the 13 subjects from Dataset 2.a, while the aggregated heart rate for the 10 subjects from Dataset 2.b is shown in Figure 5b. To avoid subject-specific differences, the heart rate was normalized using person-specific min-max normalization. Figure 5a shows that there is a linear increasing trend in the heart rate during the overall breathing session. However, that trend does not correspond to the breathing rates. To be more specific, all subjects have a minimal heart rate during the first breathing sub-session of 8 bpm, but they also have a maximal heart rate during the last breathing sub-session of 8 bpm. This is because the breathing sub-sessions are continuous (no breaks between two consecutive sub-sessions) and short (close to 60 s); thus, the changes in the heart rate caused by previous sub-sessions influence the heart rate in the following sub-sessions. Furthermore, in Figure 5b it can be seen that there are no big fluctuations in the heart rate, regardless of the breathing rates. A probable explanation for the absence of bigger fluctuations in the heart rate, which, in turn, is present in Figure 5a and in Figure 4, is the absence of more extreme breathing rates. Figure 5b contains only breathing rates between 12 bpm and 16 bpm, which are less extreme compared to the breathing rates present in Figure 5a (8–18 bpm) and Figure 4 (9 bpm–17 bpm).

## 4. Method

**Figure 6:**
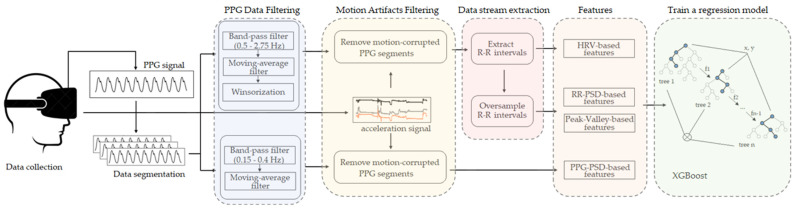
Block diagram of the proposed ML-based method for breathing rate estimation from PPG data acquired from a head-mounted device (VR headset).

This section describes our approach for automatic breathing rate estimation using PPG data. The block diagram of the proposed pipeline is shown in Figure 6. The PPG signals collected using the emteqPRO mask installed in a VR headset were firstly segmented into windows of 20 s. The segmented PPG signals were filtered using two separate filtering procedures; motion artifacts were removed from the resulting filtered signals. The filtering procedures were followed by the extraction of additional data streams based on P-P intervals. Then, we extracted four categories of features, which were designed to describe various aspects of the PPG-derived data streams. The extracted feature vectors were used to train a regression ML model, based on the extreme gradient boosting (XGBoost) algorithm. These steps are described in detail in the following subsections. The preprocessing of the data and the model training procedure was implemented using the Python programming language.

### 4.1. Data Preprocessing

Proper data pre-processing is one of the essential steps in ML methods that has a significant impact on the generalization performance of the ML models. The raw PPG signals acquired with the emteqPRO system underwent a series of preprocessing steps to convert them into an appropriate format for subsequent processing. The preprocessing steps developed as part of our method for estimation of breathing rate from PPG signals included data segmentation and filtering of the raw PPG signals, assessment of the quality of the input data and removal of motion artifacts from the PPG signals, and extraction of additional PPG-derived data streams. The data preprocessing steps are described in the following subsections.

#### 4.1.1. Data Filtering

The initial step of our breathing rate estimation procedure is the selection of an appropriate window size for segmentation of the continuous PPG signal. Previous studies related to breathing rate estimation typically employed window sizes ranging from 30 to 60 s [33]. In our work, we focused on a shorter window size that makes the algorithm more responsive to physiological changes and is more practical for real-time implementation of the algorithm. For instance, shorter windows reduce both the time required to measure the breathing rate and the computational requirements of the algorithm. Therefore, the PPG signals were segmented using a window size of 20 s, with a 1-s slide between consecutive windows.

The next step in the preprocessing pipeline is filtering of the raw PPG signals provided by the emteqPRO system. We employed two separate filtering procedures. The resulting PPG signals from the first filtering procedure were used for extraction of P-P intervals; the resulting signals from the second filtering procedure were directly utilized for feature extraction.

In the first filtering procedure, we firstly applied a third-order band-pass filter to the PPG signals to remove signal components that are mostly caused by dynamic human motion and are not related to breathing. The lower and the higher cutoff frequency were set to 0.5 and 2.75 Hz, respectively. Furthermore, the PPG signals were filtered with a moving-average filter and winsorization was applied to remove outlier values.

The second filtering procedure consists of similar steps to the first procedure. A third-order band-pass filter was applied to the PPG signals, this time with cutoff frequencies of 0.15 and 0.4 Hz. These values were selected because a normal respiratory rate in healthy people is between 9 to 24 breaths per minute. This range is even smaller for healthy adults, ranging from 12 to 20 breaths per minute [34]. The second step of this filtering procedure included a moving-average filter applied to the band-pass filtered PPG signals.

#### 4.1.2. Signal Quality Assessment and Motion Artifacts Removal in PPG Signals

**Figure 7:**
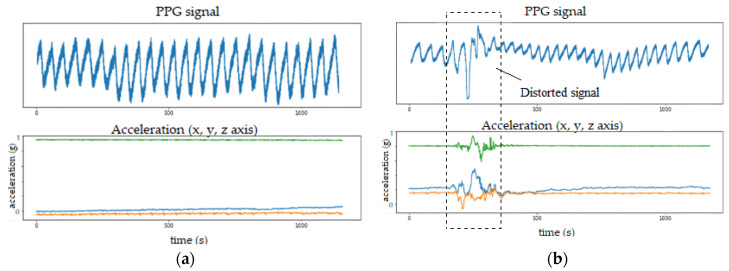
Illustration of the effect of motion on PPG signals: (**a**) clean PPG waveform when no motion is present; (**b**) PPG signal affected by motion artifacts during movement.

Even though PPG is a simple and convenient method for measuring heart activity, it is not robust to motion artifacts. When there is a lot of movement, the PPG data is usually corrupted by motion noise that appears in the form of distorted signals with large amplitudes in the signal, which are not related to heart activity. This effect is shown in Figure 7, where the PPG signal is depicted alongside a corresponding output from an accelerometer sensor (also incorporated in the emteqPRO VR mask). When no motion is present, the PPG signal form is stable (Figure 7a). However, when there is movement, the PPG signal form is altered to some extent (Figure 7b).

Motion noise can also be reflected in the frequency domain and may overlap with the frequency range of breathing activity [35]. Therefore, signal quality assessment and removal of motion artifacts present in the PPG data are vital to ensure that the proposed method will also generalize well to PPG data acquired in real-world setting during different physical activities. Therefore, we developed a signal quality assessment and motion artifacts removal procedure that utilizes accelerometer data to avoid motion-artifacts corrupted segments in the PPG signals.

To quantify the movement, we used the absolute sum of changes of the acceleration magnitude signal, calculated on 1-s window. The absolute sum of changes of a given signal is calculated as in Equation (1):

(1) $$\mathrm{absolute}\;\mathrm{sum}\;\mathrm{of}\;\mathrm{changes}=\overset{n-1}{\underset{i=0}{\sum }}\left|x_{i+1}-x_{i}\right|$$

where *x* is the vector containing the data for the specific segment, and *n* is the number of samples in the segment.

By analyzing the acceleration signals of the breathing rate sessions of each subject (from both datasets), we concluded that, in more than 90% of the recordings, there was no motion that could significantly disturb the PPG signals and interrupt the measurements. Therefore, we took the upper extreme value of the absolute sum of changes of the acceleration magnitude signals (segmented using 1-s window size) as a threshold for further analysis. Having a threshold that is based on data where the motion is limited allows us to be confident that the breathing rate is estimated using a stable PPG signal and that the derived measurements are likely to be accurate.

Once the threshold was defined, we analyzed the 20-s PPG segments alongside the corresponding acceleration signals, i.e., acceleration magnitude signals. Depending on which filtering was applied to the PPG signal (Section 4.1), we utilized the following two rules:

1. When analyzing the resulting PPG signals from the first filtering procedure (those signals are later used for extraction of P-P intervals) and their corresponding acceleration magnitude signals, we slide over the acceleration magnitude signals using a 1-s window and compare the absolute sum of changes of the particular window with the previously calculated threshold value. If the calculated value was larger than the predefined threshold, the corresponding 1-s PPG signal was removed, otherwise it was kept. The main idea here was, in a window of 20 s, to gather at least 15 s of “good” PPG signal (without motion artifacts) in order to further proceed with the extraction of P-P intervals. If the resulting PPG signal with decent quality is shorter than 15 s, we consider it as not sufficient for appropriate analysis and discard the whole 20-s window.
2. When analyzing the resulting PPG signals from the second filtering procedure (those signals are later used for frequency-domain feature extraction), we checked if short motion artifacts (1-s segments) affect the low-frequency breathing rate information contained in the PPG signal. The analysis showed that short movements, in general, result in high-frequency components and that they do not overlap with the frequency range of breathing activity. However, having multiple consecutive 1-s windows of the acceleration magnitude signal with an absolute sum of changes greater than the predefined threshold corrupted the PPG signal frequency range and the breathing rate information was lost. Therefore, we discarded the 20-s PPG segment if three or more consecutive 1-s windows had an absolute sum of changes greater than the predefined threshold unless those three consecutive windows were at the beginning or at the end of the 20-s segment.

#### 4.1.3. Data Streams Extraction

Once the filtering procedures were applied to the raw PPG signals, the next step of the data preprocessing pipeline was the extraction of additional data streams based on P-P intervals. The idea behind generating the P-P interval series was to utilize the correspondence of the P-P intervals duration with breathing activity [36]. The P-P intervals were extracted from the resulting PPG signals from the first filtering procedure. The extraction of the P-P intervals was done using our proprietary peak detection method applied on the filtered PPG signals. Additionally, in this step, a Hampel filter [37] was applied to the extracted P-P intervals series to remove outlier values. From the final P-P data stream, we also retrieved an over-sampled P-P intervals series using a quadratic interpolation function. The P-P intervals series was over-sampled to 25 Hz.

At the end of the data preprocessing procedure, we end up with three different data streams derived from the original raw PPG signal provided by the emteqPRO system: PPG filtered signal, P-P intervals time series, and an oversampled P-P intervals time series. These are then used for extraction of representative features.

### 4.2. Feature Extraction and Model Training

PPG waveforms are rich in detail and contain numerous features of interest. One of the features that are encoded in PPG signals is the breathing rate. However, there are many factors that influence the PPG signal and, as a result, the extraction of breathing rate from PPG data is not a straightforward task. Thus, we extracted a number of features, which were designed to describe the various aspects of the three PPG-derived data streams. Those features can be divided into four categories: PRV-based features, RR-PSD-based features, peak–valley-based features, and PPG-PSD-based features.

**PRV-based features:** For this group of features, we employed the regular P-P data stream, which was retrieved by detecting P-P intervals in the filtered PPG signals. We extracted general PRV features, which provide information related to breathing activity, including standard deviation of the P-P intervals, average increasing time of the P-P intervals, median duration of the P-P intervals, and the number of peaks per second.

**RR-PSD-based features:** This set of features was calculated from the spectral representation of the P-P intervals. The spectral representation captures the periodicity of the P-P intervals that is related to breathing activity. To derive the spectral representation of the P-P intervals, we calculated the PSD using Welch’s method. Given that Welch’s method assumes equidistant measurements when estimating the PSD, the P-P intervals were first oversampled to 25 Hz using a quadratic interpolation function. The PSD estimates the power distribution of an input signal over a specific frequency range, in our case between 0 and 12.5 Hz. Based on our previous work [38], we calculated the following features using the PSD as an input signal:

- Five dominant frequencies and their normalized amplitude.
- Binned distribution—shows the distribution of the FFT magnitudes into 10 equal sized bins ranging from 0 Hz to 2 Hz.

**Figure 8:**
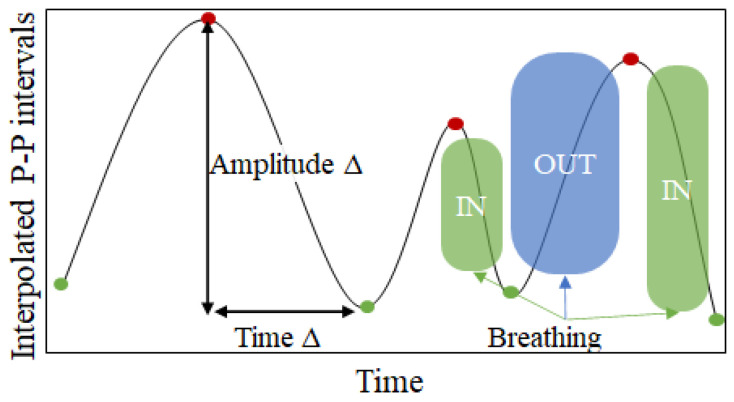
Peak–valley features from interpolated P-P intervals.

**Peak–valley-based features:** Given that with inhalation the heart rate increases (leading to shorter P-P duration) and that during exhalation the heart rate decreases (leading to longer P-P duration) [39], periodic changes are noticeable in the interpolated P-P intervals (see Figure 8). While these periodic changes are already captured by the PSD-based features, one additional way to capture this relation is to calculate the distances between the peaks (red points in Figure 8) and the valleys (green points in Figure 8) in the interpolated P-P intervals series. For each window we calculate three different metrics:

1. Amplitude delta—calculated as the amplitude change between a peak and the corresponding valley after the peak.
2. Duration delta—calculated as the distance in time between a peak and the corresponding valley after the peak.
3. Speed delta—calculated as the amplitude delta divided by the time delta.

These three metrics are calculated for each peak available in the input window. Since the number of peaks would be variable in a fixed 20-s window, we calculate aggregating statistics (median, minimum and maximum values) over all the values for one metric. Thus, we end up with 9 features (3 metrics aggregated with 3 statistical descriptors).

**PPG-PSD-based features:** This group of features was extracted from the spectral representation of the filtered PPG signals generated after the second filtering procedure. Given that, in these signals, we isolated only the frequencies that are related to breathing, the spectral representation here reflects the periodicity of the PPG signals that is related to breathing rate [40]. These features included the five dominant frequencies and their normalized amplitude, as well as the FFT magnitudes binned distribution.

Eventually, those features are fed to a regression ML-model, based on the extreme gradient boosting (XGBoost) algorithm [41], which uses decision trees as base estimators. Each tree maps the input data to one of its leaves that contains a continuous score. The training proceeds iteratively, adding new trees that predict the residuals or errors of prior trees that are then combined with previous trees to output the final prediction, which, in our case, is the estimated breaths per minute.

## 5. Experimental Setup

### 5.1. Baselines

To provide benchmark results and confirm the performance of our proposed method, we employed three baseline methods:

- Dominant frequency in the PPG signal—A baseline method that finds the dominant frequency in the raw PPG signal and calculates the breathing rate (in breaths per minute) as in Equation (2):

breathing_rate = 60/(1/dominant_PPG_frequency)(2)

- HeartPy—A baseline method from an open–source Python library [42] that estimates breathing rate from P-P intervals data. It over-samples the P-P interval series by interpolation and finds the dominant frequency in the over-sampled P-P interval series. The breathing rate (in breaths per minute) is then calculated as in Equation (3):

breathing_rate = 60/(1/dominant_RR_frequency)(3)

- DL approach—An end-to-end DL approach based on residual network architecture proposed by Bian et al. [28]. This method takes raw PPG data as input, learns the rules through training, and produces breathing rate estimation. The training of the DL model was done by using additional synthetic PPG data as described in the original study.

### 5.2. Validation and Evaluation Metrics

A leave-one-subject-out (LOSO) cross-validation technique was used to measure the performance of the proposed ML method. The LOSO cross-validation procedure is repeated N iterations, where N is the number of subjects in the dataset. In each iteration, data from one subject are used to test the model, while data from the remaining subjects are utilized to train the model. This procedure is repeated until all subjects’ data have been used as testing data. This cross-validation technique eliminates the possibility of the model learning the subject’s identity by ensuring that data from one subject is not included into both the train and test sets. As a result, the evaluation is person-independent, i.e., it simulates how the model will behave in practice—on subjects that are not included in the training process.

We report two evaluation metrics: mean absolute error (MAE), which shows how close the model’s output values are to true values, directly interpretable in terms of the unit in question—breaths per minute (Equation (4)); and Pearson’s correlation coefficient (PCC), which measures the linear correlation between the model’s output values and the true values (Equation (5)).

(4) $$\mathrm{MAE}=\frac{1}{n}\sum_{i}^{n}\left|y_{predicted}-y_{true}\right|$$

(5) $$\mathrm{PCC}=\frac{\sum_{i}^{n}\left(y_{true_{i}}-{\bar{y}}_{true}\right)\left(y_{predicted_{i}}-{\bar{y}}_{predicted}\right)}{\sqrt{\sum_{i}^{n}{\left(y_{true_{i}}-{\bar{y}}_{true}\right)}^{2}\sum_{i}^{n}{\left(y_{predicted_{i}}-{\bar{y}}_{predicted}\right)}^{2}}}$$

## 6. Results

The performance of the proposed method for breathing rate estimation was tested through a series of experiments, and the results are presented in this section. Section 6.1 presents the results obtained with the proposed method using a window of 20 s. The performance of the proposed method is also compared against two other comparison methods. Section 6.2 shows the impact of the data window size on the performance of the proposed method. Lastly, in Section 6.3, we analyze the importance of different feature sets and their influence on the performance of the proposed method. All results presented in this section are obtained using LOSO cross-validation.

### 6.1. Comparison with Baselines

This section describes the evaluation results obtained on the datasets described in Section 3. We compared the results obtained on three different combinations of data. Namely, we performed a LOSO cross-validation on the subjects’ data from Dataset 1 only, from Dataset 2 only, as well as on data from those two datasets combined. Table 1 presents the results obtained with the proposed method, as well as the results obtained with the three baselines. As described in Section 4.1, the performance of each method was obtained using a window size of 20 s.

Our proposed method outperforms the baseline methods, for all three dataset combinations, in terms of both MAE and PCC. Furthermore, it shows comparable results on all three dataset combinations, which suggests that the approach is stable and the inclusion of data from new subjects can even improve the results. When using the combination of both datasets for evaluation (LOSO on 37 subjects in total), with the proposed method, we achieve an MAE of 1.4 bpm, and a PCC of 0.8, indicating that it can easily adapt to changes in the breathing rate scenario. The high correlation score is particularly important when analyzing breathing rate during everyday activities. The range of breathing rate during everyday activities is relatively short (between 12–20 bpm in healthy adults), so even a dummy regressor that always outputs the same average value as an estimation might show low MAE. However, since it is not sensitive to changes in the breathing rate, it will produce an extremely low PCC. Such behavior can be observed when the algorithms are evaluated on Dataset 2. If we compare the results from our method and the dominant frequency approach, the MAE difference is only 0.41 bpm, but the PCC achieved with our method is almost twice as high.

Another thing that can be observed from the results presented in Table 1 is that the method that is based on P-P data (HeartPy) outperforms the method that is based on raw PPG data (dominant frequency in the PPG signal) in terms of MAE, when evaluated on Dataset 1. However, the later produces lower MAE when evaluated on Dataset 2. This suggests that both approaches are able to estimate the breathing rate to some extent, however, they are not robust enough to be used in situations when larger fluctuations in the breathing rate can be expected. This outcome supports our idea that an ML-based approach that can combine multiple sources of information is more effective for accurate estimation of breathing rate. The DL approach performs well when evaluated on Dataset 2; however, the results obtained for Dataset 1 in terms of MAE are 1.08 higher than the results obtained with our proposed method. Originally, the DL approach used a window size of 60 s, so we suspected that having three-times-shorter window size might be a huge limitation to accurately capture the breathing content. We tested the performance of the DL approach using the proposed window size of 60 s and the results show 2.15 MAE for Dataset 1, 1.13 MAE for Dataset 2 and 1.87 MAE for both datasets combined. This proved our theory that the performance of the DL approach is highly dependent on the window size.

**Figure 9:**
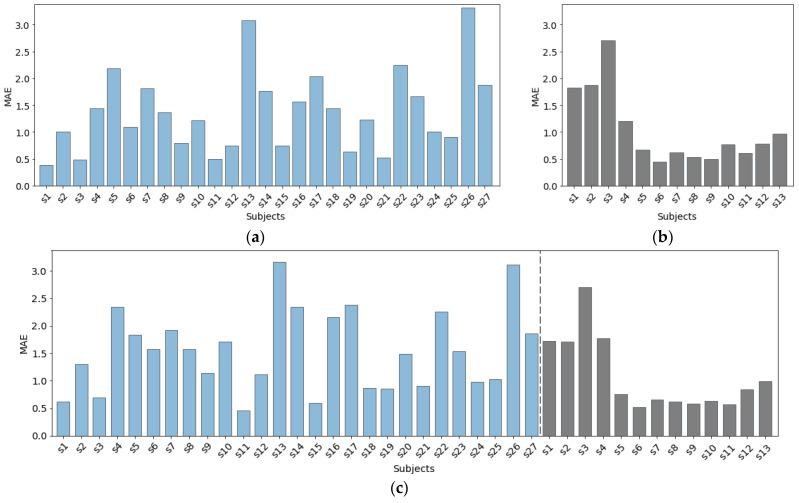
Average mean absolute error (MAE) per subject, interpretable in breaths per minute, achieved with the proposed method using leave-one-subject-out (LOSO) cross-validation on: (**a**) Dataset 1; (**b**) Dataset 2; (**c**) Dataset 1 + Dataset 2.

Figure 9 presents the MAE per subject, achieved with our proposed method for the three dataset combinations presented in Table 1. The results for Dataset 1 (Figure 9a) show noticeable variation of the MAE between different subjects. For most of the subjects (17 out of 27), the method achieves an MAE below 1.5 bpm. However, for five subjects, the predictor performed poorly, achieving an MAE higher than 2 bpm. The main reason for this was the noisy PPG data obtained during the data collection procedure.

In Dataset 2, there is a significant difference between the scores achieved for the first three subjects (Dataset 2.a) and the remaining subjects (Dataset 2.b), as seen in Figure 9b. The main reason for this outcome is that these three subjects completed five 1-min breathing sessions with a constant rate of 8, 12, 18, 12, and 8 breaths per minute, in that specified order. This scenario is different from the scenario the remaining 10 subjects were following during the data collection procedure (five 2-min breathing sessions with a constant rate of 12, 14, 16, 14, 12 bpm, in that specified order). Consequently, the model did not have enough data to learn the edge cases—the extremely low breathing rates of 8 bpm, as well as the higher breathing rate of 18 bpm. Another explanation for the higher MAE relates to the physiological relationship between the breathing rate and the PPG signal. As explained in [43], breathing rate that is out of the normal range for healthy adults (12–20 bpm) might affect the breathing rate information encoded in the PPG signal. Figure 9c presents the results achieved when data from both datasets combined was used for evaluation. The inclusion of new subjects’ data did not bring any significant improvement in terms of MAE per subject. Again, for the three subjects from Dataset 2.a, we get similarly high MAE, even though, with the inclusion of data from Dataset 1, we include more extreme values of the breathing rate in the training process. This suggests that PPG data during these breathing scenarios is noisy.

### 6.2. Influence of Window Size

Based on the analysis presented in [33], most related studies used a window size between 30 and 90 s for estimation of breathing rate from PPG signals. A multitude of studies have investigated the impact of window size on performance [22,23,44,45]; however, there is not yet a generally accepted window size that is known to be optimal for the topic at hand. We explored how our method is influenced by the employed window size and the experimental results are presented in this section.

Previous studies have shown that using a longer window leads to higher accuracy of the algorithms, which is useful since it allows for a sufficient number of breaths to be taken before the breathing rate can be reliably calculated. Moreover, it is shown that longer windows increase the range of detectable breathing rates [22]. However, there are also numerous advantages to using shorter windows, especially from a practical point of view. Namely, shorter windows reduce both the time required to measure breathing rate and the computational requirements of the breathing rate algorithms. Additionally, the use of shorter windows increases the likelihood that the breathing rate is stable throughout the window. This is especially important when the breathing rate is estimated during physical activities with high-intensity motion, since the PPG signal can be easily affected by physical movement [27].

In this experiment, we analyzed shorter windows since the added value of the breathing rate algorithm that works with such windows is higher. We tested four different window sizes, namely, 15, 20, 25, and 30 s, and the obtained results are shown in Table 2. The results show that, in contrast to the results provided in the related literature, our method does not benefit from longer windows. In fact, it performs worst in terms of both MAE and PCC when using the longest of the tested window sizes, 30 s, on all three dataset combinations. Our method performs best with the proposed window size of 20 s and these results are consistent on all three dataset combinations.

The only method that benefits from the larger window size on both datasets is the method that is based on P-P intervals data (HeartPy), especially in terms of MAE. It achieves an MAE of 2.24 bpm when using a window size of 30 s, while it achieves the lowest MAE of 2.82 when using the shortest of the tested window sizes, 15 s. These results suggest that methods that rely only on P-P intervals data for breathing rate estimation are not suitable for conditions when faster response to physiological changes is required. The optimal window size for the method that is based on raw PPG data (dominant frequency in the PPG signal) is also 20 s. It provides the lowest MAE and the highest PCC when using a 20-s window, on all three dataset combinations. Based on the results obtained with the DL approach, it can be clearly seen that, when working with Dataset 1, the MAE improves as the window size increases. However, for Dataset 2, we can see that window size of 20 s results in the best performance. An interesting observation when working with Dataset 2 is the fact that three out of four algorithms perform best with a window size of 20 s.

### 6.3. Feature Importance

In this section, we provide insight into the features that were identified as being most important for breathing rate estimation. Firstly, we investigated the performance of the method using features from each feature group described in Section 4.2, individually, to explore which category of features is the most informative. This information is also valuable for a real-time implementation of the algorithm. For instance, if better or comparable results can be achieved with only one group of features, this can reduce the computational time, as well as the computational resources required by the algorithm. The experimental results are presented in Table 3.

The results suggest that from the four groups of features that we extracted, the PPG-PSD features are the most informative for breathing rate estimation, when used independently. In fact, the ML-model trained with only this group of features achieved the lowest MAE, as well as the highest PCC for all three dataset combinations. However, if we compare these results with the results achieved when all four groups of features are used for training the ML model, it can be seen that their fusion significantly improves the results, in terms of both MAE and PCC. Namely, the MAE score is decreased for 0.29–0.36 bpm, and the PCC is increased for 0.6–0.9, for all three dataset combinations. This comparison further proved the usefulness of the proposed method that fuses various aspects of PPG-derived data streams, represented by the different feature groups.

**Figure 10:**
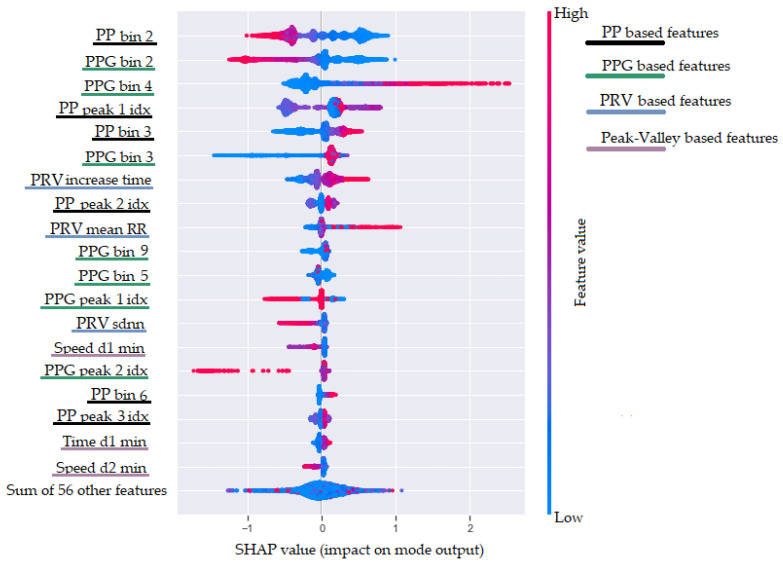
SHAP feature importance measured as the mean absolute Shapley values.

Furthermore, we tried to interpret our ML model for breathing rate estimation and to explore which features have the highest impact on the model’s output. Usually, when using a XGBoost model, the obvious choice is to use the built-in function that offers three different options for measuring the feature importance (weight, cover, and gain). However, when they are compared, the results show a relatively high difference [46]. As a result, it is difficult to choose which feature importance technique to rely on. Therefore, we decided to use the SHAP method [47], which provides advantages such as global and local interpretability, consistency, and accuracy. In addition, the SHAP values can show in which direction a particular feature is contributing to the target variable. To achieve this, the SHAP method calculates the importance of a feature by comparing what a model predicts with and without the feature. However, since the order in which a model sees features can affect its predictions, this is done in every possible order, so that the features are fairly compared. The results obtained with the SHAP method are shown in Figure 10. This plot is designed to display an information-dense summary of how the top features provided to the model impact its output. In Figure 10, we have the top 20 most informative features sorted in descending order. Each instance in the dataset is shown as one dot on each row. Furthermore, the position of the dot on the *x*-axis shows the impact of the feature on the model’s prediction. In fact, the horizontal location shows whether the effect of that value is associated with a higher or lower prediction. Additionally, the color (from blue to red) provides information about the original value of the features. Finally, we added four colors in the legend on the right of the plot to show in which of our defined feature categories each of the presented features belongs.

Figure 10 shows that the two most informative features for the ML model are *RR bin 2* and *PPG bin 2.* These features are based on the binned distribution of the FFT magnitudes of the oversampled P-P intervals stream (Section 4.1.3) and the filtered PPG stream (provided by the second filtering procedure, as explained in Section 4.1.1). These two features are negatively correlated with the breathing rate. This is expected because the second bin corresponds to low frequencies in the range from 0.1 to 0.2 Hz. Higher FFT magnitude in this bin corresponds to lower breathing rate, while lower values correspond to higher breathing rate. The third most informative feature is the *PPG bin 4*, showing the FFT magnitude in the fourth bin of the filtered PPG signal. This bin is associated with frequencies in the range from 0.3 to 0.4 Hz, which explains the positive correlation with a higher breathing rate. Other features that are related to higher breathing rate are the *PRV increase time* and *PRV mean P-P*. The *PRV increase time* feature confirms one of the effects of breathing activity on the PPG signal that it is known as amplitude modulation (AM) [23].

In the top-20 most informative features, there are features from all feature groups (PRV features, RR-PSD features, peak–valley features, and PPG-PSD features), yet the peak–valley features have a relatively small impact on the model’s output. Another interesting observation is that the dominant frequency feature extracted from the filtered PPG signal is not part of the 20 most informative features. Even though the dominant frequency is estimated from the largest magnitude in the FFT spectrum, it is not as informative as the binned distribution of the FFT magnitudes.

### 6.4. Influence of Motion Artifact Removal

The results presented in the previous sections are achieved using all data from the datasets, excluding the removal of the motion artifacts in the PPG data and the signal quality assessment procedure. The results presented in this section show the performance of our method when all proposed steps from the processing pipeline are employed—including the motion artifact filtering step and the assessment of the quality of the PPG signals.

Table 4 shows the percentage of rejected 20-s windows on both datasets, which, after the filtering step and the motion artifact removal, were found unfit for reliable breathing rate estimation, according to Rule 1, Rule 2, or both combined (see Section 4.1.2). The numbers are similar for both datasets—only 5–6% of the windows in both datasets were found unfit for reliable extraction of P-P intervals (Rule 1) or frequency-domain analysis (Rule 2). When both rules were considered, a total of 3% and 4.5% of the windows were rejected for the first and second dataset, respectively.

We estimate the breathing rate using three alternative models based on the output generated after the motion artifacts removal and the quality assessment procedure. Namely, when the quality of the PPG signal is considered good by both rules, we utilize the general model for estimation of the breathing rate that is trained on all feature groups. In case a particular window’s quality is confirmed only by one of the rules, we proceed with extraction of a subset of features—PRV-, PP-PSD-, and peak–valley-based features if the signal is considered reliable from rule 1, and PPG-PSD-based features if the signal is considered reliable from rule 2. Consequently, we use models trained with the particular subsets of features for the estimation of the breathing rate for those windows. Finally, if both procedures for assessing the quality of the PPG signal show that the specific segment is corrupted, breathing rate estimation for that window is not generated (this happens in 3% and 4.5% of the data in Dataset 1 and Dataset 2, respectively). The results obtained utilizing the full processing pipeline, including the motion artifacts removal and the signal quality assessment procedure, are shown in Table 5.

The achieved results demonstrate that the proposed method for removal of motion artifacts in the PPG signal and the procedure for assessing the quality of the motion-removed signals are effective in reducing the errors in breathing rate estimation from PPG signals. In fact, when utilizing these two additional steps in the processing pipeline, we obtained 0.02–0.03 bpm lower MAE for the three dataset combinations. Even though the data acquired in our datasets does not contain intensive motion, these results imply that the proposed method is more robust to noise (compared to the model used in Section 6.1) and can contribute to more accurate breathing rate estimation, especially in high-motion situations.

## 7. Conclusions

In this study, we presented a novel method for breathing rate estimation using data acquired from a head-worn VR mask equipped with a PPG sensor placed on the forehead of a subject. The data was collected from a total of 37 subjects during controlled breathing protocols. From the biometric sensors incorporated in the emteqPRO system, we used the data provided from the PPG sensor and the accelerometer sensor. We provide public access to a subset of the data used in this study (13 subjects); we hope that it will serve researchers for evaluation of the performance of breathing rate estimation algorithms in future studies. Furthermore, we believe that this dataset can be used as a benchmark for testing various approaches to estimate breathing rate in VR environments.

The proposed method is based on advanced signal processing and machine learning techniques. Since the PPG signal is affected by the breathing rate in multiple ways, we extracted and combined several features from multiple PPG-derived data streams. The features were designed to best describe the various aspects of each data stream that represent the different modulations of the PPG signal caused by breathing activity. The evaluation showed that the proposed method outperformed the existing approaches from the related work, achieving a mean absolute error of 1.4 breaths per minute and a Pearson’s correlation coefficient of 0.86. These results indicate that reliable estimation of breathing rate is possible based on PPG data acquired from a head-worn device.

Moreover, our method introduces a novel step for removal of motion artifacts from the PPG signals and assessing the quality of the PPG data. It utilizes accelerometer data to quantify movement and avoid motion-artifacts-corrupted segments in the PPG signals. This step showed effective in reducing the errors in breathing rate estimation from PPG signals—in fact, when it was included in the processing pipeline, we achieved a mean absolute error of 1.38 breaths per minute. Our method minimizes the number of discarded windows due to bad signal quality, while still estimating breathing rate only from reliable PPG signals.

The analysis on the impact of the employed window size on the model’s performance showed that our method benefits from a shorter window size (20 s), compared to the state-of-the-art approaches, making our algorithm more responsive to physiological changes. Furthermore, having a method that relies on shorter window size reduces the time required to measure breathing rate and the computational requirements of the breathing rate algorithm.

The feature importance analysis showed that the fusion of multiple feature groups showed significantly better results. This comparison further proved the usefulness of the proposed fusion of various aspects of PPG-derived data streams, represented by different feature groups.

Although the presented work has a few advantages over the existing methods for breathing rate estimation, it is also a subject to few limitations. First, the used datasets contain data collected in a relatively stationary position. As a result, an approximate estimate of the number of rejected windows during in-the-wild scenario cannot be obtained. Moreover, one of the possible limitations of the proposed method is its inability to detect out-of-distribution breathing rate. The models in this study were trained with data that covers the normal range of breathing rate; thus, it is an open question whether they will perform well for higher breathing rate. However, for our specific application, we do not see this as a major problem because VR activities are usually not very intensive, and the breathing rate of the participants is mostly in the expected normal range.

To avoid the aforementioned limitations, as a part of our future work we plan to develop and to test models with data recorded in-the-wild. Even though the researchers have proposed different approaches that consider the signal quality, usually, models developed with such data generalize better when tested in a real-life scenario. However, obtaining real-life annotated data can be quite challenging. Therefore, we plan to take advantage of unlabeled PPG data by using semi-supervised ML approaches. Moreover, creating synthetic data that include diverse levels of motion activity to mimic more realistic scenarios can be used to develop more robust ML models for breathing rate estimation. Finally, we plan to explore self-supervised learning in order to overcome the problems caused by human motion using unlabeled PPG data.

## Figures and Tables

**Table 1:** Average mean absolute error (MAE), interpretable in breaths per minute, and Pearson’s correlation coefficient (PCC) achieved with the proposed ML-method and three baseline methods, using a window size of 20 s. The results are achieved using leave-one-subject-out (LOSO) cross-validation, on three dataset combinations.

Dataset	Our Method		HeartPy		Dominant Frequency		DL Approach	
	PCC	MAE	PCC	MAE	PCC	MAE	PCC	MAE
Dataset 1	0.88	1.37	0.57	2.18	0.51	2.50	0.54	2.45
Dataset 2	0.81	1.04	0.45	2.38	0.44	1.45	0.55	1.15
Dataset 1 + Dataset 2	0.86	1.4	0.50	2.30	0.47	1.78	0.54	2.17

**Table 2:** Average mean absolute error (MAE), interpretable in breaths per minute, and Pearson’s correlation coefficient (PCC) achieved with the proposed ML-method and two baseline methods, using different window sizes. The results are achieved using leave-one-subject-out (LOSO) cross-validation, on three dataset combinations.

Dataset	Method	15 s		20 s		25 s		30 s	
		PCC	MAE	PCC	MAE	PCC	MAE	PCC	MAE
Dataset 1	Our method	0.84	1.59	0.88	1.37	0.87	1.5	0.85	1.64
	HeartPy	0.53	2.68	0.57	2.18	0.52	2.45	0.52	2.44
	Dominant Frequency	0.45	2.9	0.51	2.5	0.2	3.02	−0.33	3.46
	DL approach	0.42	2.8	0.54	2.45	0.54	2.42	0.55	2.39
Dataset 2	Our method	0.73	1.19	0.81	1.04	0.68	1.25	0.61	1.29
	HeartPy	0.35	2.91	0.45	2.38	0.44	2.13	0.44	2.11
	Dominant Frequency	0.41	1.69	0.44	1.45	0.15	2.33	0.01	3.81
	DL approach	0.39	1.54	0.55	1.15	0.58	1.21	0.47	1.47
Dataset 1 + Dataset 2	Our method	0.82	1.58	0.86	1.4	0.84	1.46	0.81	1.59
	HeartPy	0.43	2.82	0.5	2.3	0.47	2.25	0.48	2.24
	Dominant Frequency	0.43	2.06	0.47	1.78	0.2	2.55	−0.16	3.7
	DL approach	0.41	2.47	0.54	2.17	0.55	2.15	0.53	2.21

**Table 3:** Average mean absolute error (MAE), interpretable in breaths per minute, and Pearson’s correlation coefficient (PCC) achieved with the proposed ML-method trained with different feature sets, using a window size of 20 s. The results are achieved using leave-one-subject-out (LOSO) cross-validation, on three dataset combinations.

Dataset	PRV		PP-PSD		Peak–Valley		PPG-PSD		ALL	
	PCC	MAE	PCC	MAE	PCC	MAE	PCC	MAE	PCC	MAE
Dataset 1	0.31	3.34	0.78	1.76	0.38	3.08	0.82	1.73	0.88	1.37
Dataset 2	0.14	2.26	0.50	1.65	0.33	2.19	0.72	1.35	0.81	1.04
Dataset 1 + Dataset 2	0.22	3.13	0.71	2.03	0.42	2.82	0.79	1.69	0.86	1.4

**Table 4:** The percentage of rejected 20-s windows on both datasets, according to Rule 1, Rule 2, and both rules combined.

Dataset	Rule 1	Rule 2	Rule 1 and Rule 2
Dataset 1	5%	6%	3%
Dataset 2	6%	5%	4.5%

**Table 5:** Average mean absolute error (MAE), interpretable in breaths per minute, and Pearson’s correlation coefficient (PCC) achieved with and without motion artifact removal and signal quality assessment procedure (Section 4.1.2). The results are achieved using leave-one-subject-out (LOSO) cross-validation, on three dataset combinations.

Dataset	Without Filtering		With Filtering	
	PCC	MAE	PCC	MAE
Dataset 1	0.88	1.37	0.88	1.35
Dataset 2	0.81	1.04	0.82	1.01
Dataset 1 + Dataset 2	0.86	1.4	0.86	1.38

## Data Availability

The dataset used in this study can be found at: https://github.com/emteqlabs/BreathingDataVR (accessed on 7 March 2022). The dataset is solely intended for non-commercial, scientific, or educational purposes.
